# Mitochondrial Homeostasis and Mast Cells in Experimental Hepatic Ischemia-Reperfusion Injury of Rats

**DOI:** 10.5152/tjg.2022.21911

**Published:** 2022-09-01

**Authors:** Suleyman Koc, Halef Okan Dogan, Ozhan Karatas, Mehmet Mustafa Erdogan, Vural Polat

**Affiliations:** 1 Department of General Surgery, Sivas Cumhuriyet University Faculty of Medicine, Sivas, Turkey; 2 Department of Biochemistry, Sivas Cumhuriyet University Faculty of Medicine, Sivas, Turkey; 3 Department of Pathology, Sivas Cumhuriyet University Faculty of Veterinary Medicine, Sivas, Turkey; 4 Department of Histology and Embryology, Malatya Educatıon and Research Hospital, Malatya, Turkey; 5 Department of Cardiovascular Surgery, Sivas Cumhuriyet University Faculty of Medicine, Sivas, Turkey

**Keywords:** Chymase, ischemia-reperfusion, liver injury, mast cells, uridine

## Abstract

**Background:**

Ischemia-reperfusion injury is a histopathological event and is an important cause of morbidity and mortality after hepatobiliary surgery. We aimed to investigate the protective effect of uridine on hepatic ischemia-reperfusion injury in rats.

**Methods:**

he animals were divided into 4 groups (n = 8): group I (control), group II: ischemia-reperfusion (30 minutes ischemia and 120 minutes reperfusion), group III: ischemia-reperfusion + uridine (at the beginning of reperfusion), and group IV: ischemia-reperfusion + uridine (5 minutes before ischemia-reperfusion). Uridine was administered a single dose of 30 mg/kg IV. The 3 elements of the hepatoduodenal ligament (hepatic artery, portal vein, and biliary tract) were obliterated for 30 minutes. Then hepatic reperfusion was achieved for 120 minutes.

**Results:**

In the ischemia-reperfusion group, both liver tissues and serum chymase activity and high-temperature requirement A2 levels were higher. Severe central vein dilatation and congestion, widening sinusoidal range, diffuse necrotic hepatocytes and dense erythrocyte accumulation in sinusoids, and strongly inducible nitric oxide synthase expression were seen in the ischemia-reperfusion group. A clear improvement was seen in both uridine co-administration and pretreatment groups.

**Conclusion:**

Our results revealed that uridine limits the development of liver damage under conditions of ischemia-reperfusion, thus contributing to an increase in hepatocyte viability.

## Main Points

- Ischemia-reperfusion (I/R)-induced liver injury consists of a complex series of events during both ischemia and reperfusion. The mechanism of I/R injury is not yet fully understood. Little is known about the protective agents against this damage of I/R on the liver and other organs.
- In this study, we aimed to examine the hepatoprotective effect of uridine against I/R -induced liver damage and mast cells’ roles in this injury, and the hepatoprotective effect of chymase, the degranulation product of these cells.
- Our results revealed that uridine limits the development of liver damage under conditions of I/R, thus contributing to an increase in hepatocyte viability.
- Therefore, it is reasonable to conclude that uridine is a promising pharmacological “tool” for correcting histologic and metabolic changes developed under oxidative stress caused by I/R damage conditions. However, further studies are needed to understand the possible mechanisms by which uridine can prevent liver injury by I/R.

## Introduction

Ischemia-reperfusion (I/R) induced liver injury is a pathophysiological condition that occurs after trauma, resection, transplantation, and circulatory shock. Ischemia-reperfusion injury to the liver is one of the leading causes of morbidity and mortality after hepatobiliary surgery.^1^ A close association has been found between delayed graft function and I/R damage after liver transplantation.^2^

Ischemia-reperfusion damage in the liver includes 2 dynamic stages. The first stage is the ischemic process. At this stage, the decrease or absence of tissue blood supply interrupts electrons’ flow in the respiratory chain of mitochondria and inhibits oxidative phosphorylation. In adenosine triphosphate (ATP) deficiency, glycolysis accelerates lactate synthesis increases, resulting in a decrease in pH in the intracellular environment. Inhibition of Na, K-ATPase leads to water inflow to the cytoplasm and cell swelling.^3^ In the second stage, activated macrophages in the liver increase reactive oxygen species production, causing oxidative stress. The imbalance of oxidative status leads to endothelial dysfunction, DNA damage, and local inflammatory responses.^4^

Mast cells (MCs) originating from myeloid cells and migrating to peripheral tissues play an important role in initiating and regulating the inflammatory reaction. Mast cells also store and secrete active inflammatory serine proteases such as tryptase, chymase, carboxypeptidase A, and dipeptidyl peptidase to regulate the local inflammatory response. Chymase is the most notable recently.^5^ Although chymase has been the subject of many studies due to its roles mentioned above, there are very few studies on I/R-induced liver injury.

High-temperature requirement A2 (HtrA2) is a protein encoded in the nucleus and is found largely between mitochondrial membranes as well as in the endoplasmic reticulum and nucleus. High-temperature requirement A2 is involved in the maintenance of mitochondrial homeostasis.^6^ It has been found that there is a relationship between HtrA2 and certain diseases such as neurodegenerative disorders, pulmonary inflammation, rheumatoid arthritis, and regulation of cell apoptosis. However, we know little about the role of HtrA2 in liver I/R injury.^7^

Many animal studies have shown that antioxidant agents are useful in protecting against I/R-induced organ injury.^8^ Uridine, a potent-free radical scavenger, has anti-inflammatory, antioxidant, and anticancer effects.^9^ Moreover, uridine can be found free in blood and tissues and participate in biochemical events such as glycogen biosynthesis, protein, and lipid glycosylation and pyrimidinergic transmission structure of nucleotides, nucleotide sugars, nucleic acids, and normalization of the balance between the free radical oxidation intensity and the antioxidant defense.^9,10^

Despite basic and clinical research efforts, a detailed molecular mechanism of liver I/R injury and uridine effects has not been described. In this study, we aimed to examine the hepatoprotective effect of uridine against I/R-induced liver damage and the roles of chymase and HtrA2. Besides, we evaluated histopathological and immunohistochemical changes in terms of 8-hydroxy-2′-deoxyguanosine (8-OhDG) and inducible nitric oxide synthase (iNOS) expressions in the experimental I/R-induced liver injury of rats.

## Materials and Methods

### Animals

For this experimental animal study, approval was provided from Sivas Cumhuriyet University Animal Experiments Local Ethics Committee (decision no.65202830-050.04.04-349). All the procedures involving animals were performed according to the institution's ethical standards at which the studies were conducted. We used 32, 250-300 g, 10- to 12-week-old male Wistar rats obtained from the Sivas Cumhuriyet University Experimental Animal Production Application and Research Center.

### Experimental Design

The rats were divided into 4 groups, with 8 in each group. Group I: control (only laparotomy), group II: I/R (30 minutes ischemia and 120 minutes reperfusion), group III: I/R + 30 mg/kg IV uridine (Sigma-Aldrich, Beijing, China) at the beginning of reperfusion immediately after ischemia) and group IV: I/R + uridine (30 mg/kg IV 5 minutes before I/R). Rats were anesthetized 60 mg/kg sodium pentobarbital (Penbital, Bioveta, Ivanovice na Hané, Çekya) intraperitoneally. Laparotomy was performed through a median incision. In order to reach the hepatoduodenal ligament easily, the intestinal loops were protected with a wet gauze sponge and lateralized to the left. After that, the right-left and central lobes were lifted upwards for easy access to the liver hilum. The 3 elements of the hepatoduodenal ligament (hepatic artery, portal vein, and biliary tract) were obliterated by placing bulldog mini clamps for 30 minutes. Then the clamps were removed to allow hepatic reperfusion for 120 minutes.

## Biochemical Analyses

### Preparation of Liver Tissue Homogenates

The liver was thawed, weighed, and then homogenized in ice-cold 10 mL/g phosphate-buffered saline (PBS) (0.01 M, pH 7.4) (Sigma-Aldrich). A 25-g tissue sample was weighed. The homogenates were then centrifuged for 5 minutes at 4.620 × g, and the supernatant was kept at −80°C until analysis.

### Determination of HtrA2 and Chymase Concentrations

The quantitative sandwich ELISA technique was used to determine HtrA2 and chymase (Bioassay Technology Laboratory, Shanghai, China) concentrations. The tests were carried out according to the information in the manufacturer’s user guide.

### Blood Samples

After blood samples were taken, plasma fractions were separated by centrifugation (2.058 × g, 15 minutes, and 4°C). They were then split and quickly stored at −80°C (WiseCryo, Seoul, South Korea).

### Histopathology

The liver tissue samples were taken from each animal immediately after euthanasia, fixed in 10% formalin and after processed in an autotechnicon device, later embedded in paraffin blocks. The blocks were cut at 5-µm thickness, deparaffinized, rehydrated using standard techniques, and sections were stained with hematoxylin–eosin (H&E) and Masson’s-trichrome stains using standard protocols for analysis by light microscopy (Eclipse E 600; Nikon, Tokyo, Japan). A veterinary pathologist performed histological analysis. The histopathological changes in H&E stained sections were examined for cellular congestion and necrosis. The degree of hepatic injury was evaluated according to grades as follows: 0, absent; 1, mild; 2, moderate; 3, severe.

### Immunohistochemistry Staining

Sections taken from the blocks on 5-μm thick polylysine slides were stained immunohistochemically. Sections were allowed to cool in normal room conditions for 20 minutes, then washed in PBS (P4417-50TAB; Sigma-Aldrich) 3 times for 5 minutes each time. Then hydrogen peroxide was applied to the sections for 10 minutes to block endogenous peroxidase activity. The sections were treated with antigen retrieval (AR) solution at 500 W, twice for 5 minutes, to measure the tissues’ antigen. After applying Ultra V Block solution (TA-125-HP; Lab Vision Corp, Fremont, CA, USA) for 5 minutes, sections were incubated with anti-8-OhDG antibody (Santa Cruz, Cat no. sc-66036, CA, USA) and anti-iNOS antibody (Abcam, Cat. no. ab15323, Cambridge, UK) diluted 1 : 200 for 60 minutes at room temperature in a humid environment. After washing 3 times with PBS for 5 minutes each time, sections were stained with Lab Vision™ UltraVision™ Large Volume Detection System: anti-Polyvalent, horseradish peroxidase (HRP) secondary immunohistochemistry kit (Thermofisher, Cat. no. TP-125-HL, Vantaa, Finland). During this process, 3,3’-diaminobenzidine was used as a chromogen/substrate. After washing 3 times with PBS for 5 minutes each time, sections were counterstained with Mayer’s hematoxylin, passed through alcohol xylol series and covered with Stellan, and examined under the light microscope. The degree of immune positivity was evaluated according to grades as follows: 0, absent; 1, mild; 2, moderate; 3, severe.

### Immunofluorescence Staining

For this purpose, 5-µm sections taken on polylysine slides were passed through the loll and alcohol series. The sections were treated with AR solution at 500 W, twice for 5 minutes, to measure the tissues’ antigen. After washing 3 times with PBS for 5 minutes each time, sections were incubated with anti-8-OhDG primary antibody (Santa Cruz) and anti-iNOS primary antibody (Abcam) diluted 1 : 200 for 60 minutes at room temperature in a humid environment. After washing the tissues with PBS again, they were treated with secondary antibodies attached to fluorescein isothiocyanate (FITC) (goat anti-Mouse FITC, mouse anti-Rabbit FITC, St John’s Laboratory, Darmstadt, Germany) at 37°C for 45 minutes. After washing 3 times with PBS for 5 minutes each time, 4,6-diamidino-2-phenylindole dihydrochloride (Merc, Darmstadt, Germany) was dropped onto the tissues and examined under the fluorescence microscope. The degree of immunofluorescence positivity was evaluated according to grades as follows: 0, absent; 1, mild; 2, moderate; 3, severe.

### Statistical Analysis

Biochemical, histopathological, immunohistochemical, and immunofluorescence examination results were analyzed with Statistical Package for the Social Sciences version 20.0 software program (IBM Corp.; Armonk, NY, USA). Biochemical data were evaluated with a one-way analysis of variance and post hoc Tukey tests. For histopathological findings, the difference between the groups was evaluated by Kruskal–Wallis, which is one of the nonparametric tests, and the group that made the difference with the Mann–Whitney U-test (*P* < .05).

## Results

### Biochemistry

In order to evaluate the liver oxidative stress-mediated liver injury, we measured the levels of chymase and HtrA2 in liver tissues and serum. Comparison of laboratory parameters among groups was shown in Table 1 and Table 2, respectively. Both liver tissues and serum chymase activity were increased in group 2 (I/R) when compared to the control group (*P* < .001). No difference was determined between group 1 (control) and 3 in terms of chymase. Lower chymase activity was found in group 3 (uridine was administered at the beginning of reperfusion immediately after ischemia) and group 4 (uridine administered 5 minutes before the I/R) compared to group 2 (I/R) (*P* < .001). The lowest chymase level was detected in group 4. The mean liver tissues and serum HtrA2 levels were statistically significantly higher in group 2 (I/R) compared to the control group. Moreover, statistically, significantly lower HtrA2 activity was found in group 3 and group 4 compared to group 2 (I/R).

### Histopathologic Findings

**Figure 1.**
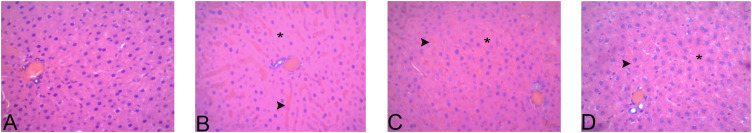
Representative micrographs showing the liver samples of all experimental groups. Control group (A) shows normal liver architecture. Ischemia-reperfusion (I/R) group (B) shows severe necrotic hepatocytes (*) and congestion (arrowhead) compared to the control group. Section from a rat of group III: I/R + uridine (before reperfusion) (C) shows moderately necrotic hepatocytes (*) and congestion (arrowhead) compared to group II. Group IV: I/R + uridine (5 minutes before I/R) (D) showing mild necrotic hepatocytes (*) and congestion (arrowhead) compared to group II. ×20- hematoxylin–eosin staining

The classic hepatic structure was shown in control group I (Figure 1A). Ischemia-reperfusion-induced liver injury was associated with prominently dilatation and congestion of the central vein (CV), severe congestion of the sinusoidal spaces (arrows), and diffuse necrotic hepatocytes around the CV and dense erythrocyte accumulation in sinusoids (Figure 1B). However, in the uridine co-administration and pretreatment groups (groups 3 and 4), it was observed that the CV was mildly dilated with minimal congestion, and the hepatocytes were located around the sinusoidal space normally (arrows) (Figure 1C, D). It was observed that this improvement mentioned above was greater in group 4 compared to group 3 (Table 3).

### Immunohistochemical and İmmunofluorescent Findings

**Figure 2.**
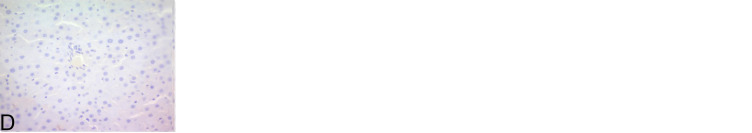
Representative micrographs from group I (A), group II (B), group III (C), and group IV (D) shows 8-hydroxy-2’-deoxyguanosine immune negativity. ×20 immunohistochemical staining.

**Figure 3.**
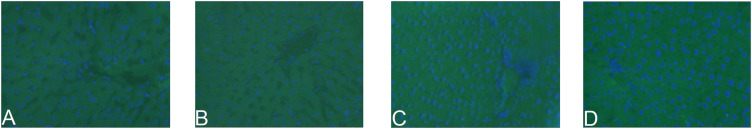
Representative micrographs from group I (A), group II (B), group III (C), and group IV (B) shows 8-hydroxy-2’-deoxyguanosine immune negativity. ×20 immunofluorescent staining.

**Figure 4.**
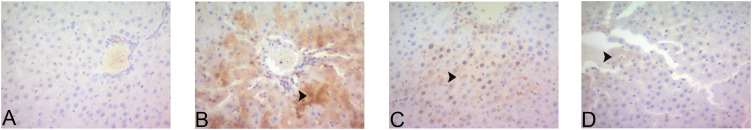
Representative micrographs showing iNOS expression for all experimental groups. Control group I (A) showing İNOS immune negativity. Ischemia-reperfusion group II (B) shows severe iNOS expression (arrowhead) compared to the control group. Group III (C) shows moderately iNOS expression (arrowhead) compared to group II. Group IV (D) shows mild iNOS expression (arrowhead) compared to group II. ×20 immunohistochemical staining.

**Figure 5.**
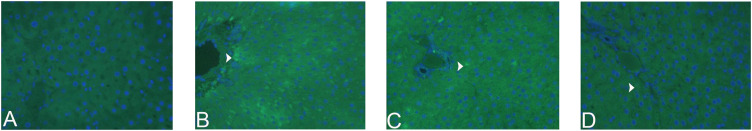
Representative micrographs showing iNOS expression for all experimental groups. Control group I (A) showing İNOS immune negativity. Ischemia-reperfusion group II (B) shows severe iNOS expression (arrowhead) compared to the control group. Group III (C) shows moderately iNOS expression (arrowhead) compared to group II. Group IV (D) shows mild iNOS expression (arrowhead) compared to group II. ×20 immunofluorescent staining.

When the staining results performed by immunohistochemical and immunofluorescence methods were compared, the 8-OhDG expression rates of the groups were similar. (Figure 2-3), while statistically significant differences were detected between the groups in terms of iNOS positivity (Table 3). While no iNOS immunopositivity was found in control rats’ livers, it was strongly iNOS expression found in group II’s hepatocyte cytoplasm. Group II had severe immunity among the experimental groups, while it was statistically significantly moderate in group III and group IV (Figure 4-5).

## Discussion

Ischemia-reperfusion-induced liver injury consists of a complex series of events during both ischemia and reperfusion.^3^ The mechanism of I/R injury is not yet fully understood. Little is known about the protective agents against this damage of I/R on the liver and other organs. Thus, we aimed to investigate the hepatoprotective effect of uridine against I/R-induced liver injury. Our study demonstrated that uridine effectively reduced I/R-induced histopathological changes such as congestion, necrosis, and iNOS expression in the liver. Moreover, it reduced the levels of chymase and HtrA2. Our study revealed that uridine could protect the liver against I/R-induced hepatic injury in a rat model.

Pringle maneuver is often used during liver surgery to reduce unnecessary blood loss. However, this maneuver causes I/R injury in the liver.^11^ Due to the depletion of ATP during ischemia, cell membrane stabilization is impaired, and Na^+^, Ca^2+^, and hydrogen accumulate into the cell, causing cell swelling due to hyperosmolarity.^3^ During I/R, the CV dilatation and mononuclear cell infiltration occur. Moreover, the neutrophils are involved in the process and release inflammatory mediators that can cause hydropic degeneration, congestion, and cell death during reperfusion.^12-14^ Our study revealed that I/R injury was associated with markedly dilatation and congestion of the CV and sinusoidal spaces, diffuse necrotic hepatocytes around the CV, and erythrocyte accumulation in sinusoids. However, hepatic histological changes were markedly reduced in the uridine co-administration or pretreatment groups.

Mast cell resides around blood vessels in the liver. Damage to liver sinusoid endothelial cells causes inflammatory cells to move into the liver.^15^ In previous studies found that a close relationship has been found between the damage caused by I/R and the activation and degranulation of MCs.^16,17^ Mast cells store and release various active inflammatory serine proteases such as tryptase, chymase, carboxypeptidase A, and dipeptidyl I during I/R. Previous studies also reported that chymase triggers cellular proliferation, migration, activation, and cytokine production.^18,19^ Since chymase plays an important role in the mechanisms of inflammation, allergy, angiogenesis, oncogenesis, and changes in organ cytoarchitectonics, it should be considered as a diagnostic marker in pathological conditions.^20^ It has been suggested that inhibition of MCs degranulation with drugs at an early stage may induce a decrease in an inflammatory response, thus alleviating I/R-induced organ damage.^16,21^ In our study, we found that chymase was at a higher level in the I/R group compared to the control and given uridine together, groups show that chymase also plays a role in inflammation, and uridine has a positive effect against this inflammation by reducing chymase levels. Thus, there appears to be a close relationship between chymase expression and inflammation.

High-temperature requirement A2 is a protein encoded in the nucleus found largely in the space between mitochondrial membranes and the endoplasmic reticulum and nucleus. It has been reported that HtrA2, released from mitochondria to cytosol, interacts with proteins that inhibit cell death and acts to prevent caspase inhibition.^6,7^ In studies conducted, it has been stated that the function of HtrA2 is variable. It supports cell survival by maintaining mitochondrial homeostasis under normal conditions, while it stimulates cell death rather than a protector in cases of oxidative stress.^22,23^ In this study, we found that HtrA2 expression due to I/R injury, which is accepted as a stress condition, was significantly increased in rats’ liver tissues and serum, and a decrease in the I/R and given uridine together groups (III and IV). We believe that this is due to the protective effect of uridine against oxidative stress damage in the liver caused by I/R.

Inducible nitric oxide synthase, which produces nitric oxide (NO) from l-arginine, participates in many inflammatory and immune events. Although iNOS is necessary for normal physiology, over-expression or dysregulation of iNOS causes excessive NO production, which plays a role in developing some human diseases.^24^ A previous study found that uridine given before I/R exerts a protective effect on mouse livers by increasing the hepatocytes’ tolerance to hypoxia.^25^ In another study supporting this, a positive relationship was found between hypoxia and the increase in iNOS level.^26^ Similar to previous studies’ results, in our study, we immunohistochemically and immunofluorescent found that iNOS expression in the I/R group was the highest compared to the other groups. Our results revealed that iNOS mediates acute I/R-induced liver injury and that uridine inhibition provides beneficial effects in preventing acute liver injury.

Although many different oxidative damage products have been identified, 8-OHdG is an important marker defined many years ago in DNA damage.^27^ Therefore, in our study, 8-OhDG expression was immunohistochemically and immunofluorescent examined in terms of DNA damage, but no difference was found between the I/R group and the groups treated with uridine in terms of 8-OHDG expression. This situation is attributed to the shortness of the I/R time or the role of a different I/R-induced oxidative stress pathway.

It has been found in previous studies that uridine has been shown to increase mouse liver hepatocytes’ tolerance to hypoxia.^25,28^ We observed that in the I / R and given uridine together groups (III and IV), decreased biochemical and histopathological changes were observed compared to the I/R group. When comparing these 2 groups, group IV’s greater improvement (urine has given 5 minutes before I/R) compared to group III (uridine given at the end of ischemia) indicates that prior administration for prophylactic purposes may have a higher antioxidant effect. It is thought that this situation may be related to the metabolism of uridine. In the present study, the uridine was observed to exhibit hepatoprotective effect as demonstrated by a significant decrease in both tissue and serum chymase and HtrA2 level in rat I/R-induced liver injury. Moreover, reduced histological damage such as congestion, necrosis, and a significantly decreased iNOS expression in liver tissue suggests that the reduction of oxidative stress in this scenario likely plays a role in the hepatoprotective effects of uridine.

In this study, we demonstrated the promising effect of uridine in correcting the histopathological and metabolic changes of I/R-mediated oxidative stress damage, which inevitably occurs in hepatobiliary surgery, especially through the reduction of chymase, which is the degranulation product of MCs. These findings may encourage using drugs such as ketotifen, indanone, cromolyn sodium, quercetin, and luteolin, which are already used as MCs stabilizers, as prophylactic agents against I/R-mediated liver damage.^29,30^

## Conclusion

Our results revealed that uridine, administered to rats intravenously before I/R or at the beginning of reperfusion, limits the liver’s damage development under conditions of I/R, thus contributing to an increase in hepatocyte viability. Therefore, it is reasonable to conclude that uridine is a promising pharmacological “tool” for the correction of histologic and metabolic changes developed under oxidative stress caused by I/R damage conditions. However, further studies are needed to understand the possible mechanisms by which uridine can prevent liver injury by I/R.

## Figures and Tables

**Table 1.** Comparison of Liver Homogenates Chymase and HtrA2 Levels Between Groups

Parameters	Experimental Group				*P*
Control Group	Group II	Group III	Group IV
**Chymase (ng/mL)**	0.87 ± 0.4^a^	1.33 ± 0.46^b^	0.85 ± 0.39^a^	0.53 ± 0.18^c^	<.001
**HtrA2 (ng/mL) **	115.38 ± 15.54^a^	239.92 ± 57.76^b^	183.87 ± 17.29^c^	152.85 ± 13.56^d^	<.001

Results were given as mean ± standard deviation (n = 8 for each group).

Different upper superscripts indicate statistical differences among groups (*P* < .001).

HtrA2, high-temperature requirement 2.

**Table 2.** Comparison of Blood Chymase and HtrA2 Levels Between Groups

Parameters	Experimental Group				*P*
Control Group	Group II	Group III	Group IV
**Chymase (ng/mL)**	0.75 ± 0.38^a^	1.21 ± 0.52^b^	0.78 ± 0.27^a^	0.51 ± 0.23^c^	<.001
**HtrA2 (ng/mL) **	106.38 ± 14.67^a^	218.78 ± 43.16^b^	147.25 ± 16.23^c^	118.62 ± 17.05^d^	<.001

Results were given as mean ± standard deviation (n = 8 for each group).

Different upper superscripts indicate statistical differences among groups (*P* < .001).

HtrA2, high-temperature requirement 2.

**Table 3.** Comparison of Histopathological Findings for All Groups

Experimental Group	Congestion/Necrosis
Group I	0.16 ± 0.40^a^
Group II	2.66 ± 0.51^b^
Group III	1.83 ± 0.40^c^
Group IV	1.16 ± 0.40^d^

Results were given as mean ± standard deviation (n = 8 for each group).

Different upper superscripts indicate statistical differences among groups (*P* < .05).

**Table 4. t4-tjg-33-9-777:** Comparison of İmmunohistochemical and İmmunofluorescent Findings for All Groups

Parameters	Experimental Group				*P*
Control Group	Group II	Group III	Group IV
**iNOS**	0.33 ± 0.51^a^	2.83 ± 0.40^b^	2.16 ± 0.40^c^	1.33 ± 0.51^d^	<.05
**8-OhDG**	0.16 ± 0.40^a^	0.16 ± 0.40^a^	0.16 ± 0.40^a^	0.16 ± 0.40^a^	

Results were given as mean ± standard deviation (n = 8 for each group).

Different upper superscripts indicate statistical differences among groups (*P* < .05).

8-OHdG, 8-hydroxy-2′-deoxyguanosine; iNOS, inducible nitric oxide synthase.
